# Untangling the evolution of body-part terminology in Pano: conservative versus innovative traits in body-part lexicalization

**DOI:** 10.1098/rsfs.2022.0053

**Published:** 2022-12-09

**Authors:** Roberto Zariquiey, Javier Vera, Simon J. Greenhill, Pilar Valenzuela, Russell D. Gray, Johann-Mattis List

**Affiliations:** ^1^ Pontificia Universidad Católica del Perú, Lima, Peru; ^2^ School of Biological Sciences, University of Auckland, Auckland, New Zealand; ^3^ DLCE, Max Planck Institute for Evolutionary Anthropology, Leipzig, Sachsen, Germany; ^4^ World Languages & Cultures, Chapman University, Anaheim, CA, USA

**Keywords:** body-part nouns, language-family specific traits, linguistic phylogeny, lexicalization, Pano languages, amazonian languages

## Abstract

Although language-family specific traits which do not find direct counterparts outside a given language family are usually ignored in quantitative phylogenetic studies, scholars have made ample use of them in qualitative investigations, revealing their potential for identifying language relationships. An example of such a family specific trait are body-part expressions in Pano languages, which are often lexicalized forms, composed of bound roots (also called body-part prefixes in the literature) and non-productive derivative morphemes (called here body-part formatives). We use various statistical methods to demonstrate that whereas body-part roots are generally conservative, body-part formatives exhibit diverse chronologies and are often the result of recent and parallel innovations. In line with this, the phylogenetic structure of body-part roots projects the major branches of the family, while formatives are highly non-tree-like. Beyond its contribution to the phylogenetic analysis of Pano languages, this study provides significative insights into the role of grammatical innovations for language classification, the origin of morphological complexity in the Amazon and the phylogenetic signal of specific grammatical traits in language families.

## 1. Introduction

**Figure 1.**
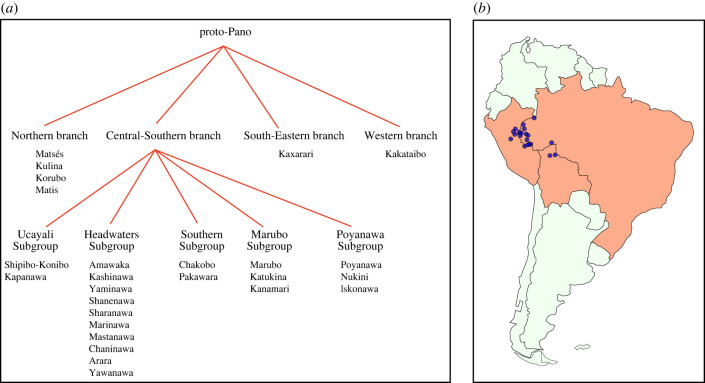
Internal classification of Pano languages based on Valenzuela & Guillaume [5] (figure 1*a*) and approximate location of the Pano languages in our sample (figure 1*b*).

Pano is a language lineage of Western Amazonia. It comprises approximately 33–34 (extant and dormant) languages from neighbouring territories in eastern Peru, western Brazil and northern Bolivia. There have been various internal classification proposals for the Pano language family in the literature, but there is no full agreement on the structure of the Pano phylogenetic tree, the classification of some languages, and the number of major branches [1–5]. This paper takes Valenzuela & Guillaume's [5] classification (presented in figure 1*a*), as a reference point for the analyses presented in the following sections, but a definitive Pano phylogenetic classification is still to be done.

Pano languages exhibit a significant list of shared grammatical features, which may be suggesting a shallow time-depth [3]. Among these shared features, which are fundamental for understanding the evolution of the Pano language family, are some salient properties associated with body-part expressions. Pano languages often exhibit an interesting and widespread morphological pattern regarding their body-part terminology, according to which body-part nouns tend to exhibit a diachronic morphological structure composed by monosyllabic bound roots and a closed set of non-productive derivative morphemes (morphological formatives), most of which are semantically opaque. These combinations of body-part roots and body-part formatives are lexicalized items in the sense that, independently of their likely morphologically complex origin, they should synchronically be analysed as simple lexical items. Body-part roots can be easily identified as in most languages they also operate as synchronically productive ‘body-part prefixes’, and as so may attach to nouns, verbs and adjectives [6].

For example, in Kakataibo, the lexicalized noun for ‘hand’ is *mɨkɨn*, which is synchronically non-segmentable, but can be diachronically analysed as the combination of the root *mɨ*- ‘hand’ and the formative -*kɨn.* The root *mɨ-* ‘hand’ can also function as a prefix and as such it can be attached to nouns (*mɨ-ʂaká* ‘skin located on the hands' [<*ʂaká* ‘skin’], adjectives (*mɨ-tunan* ‘black-handed’ [< *tunan* ‘black’]), and verbs (*mɨ-táʃka* ‘to slap on the hand’ [< *táʃka* ‘to slap’]). Many expressions related to external body-parts exhibit a similar pattern: Iskonawa *tɨhu* ‘neck’ (*tɨ-hu*), Kapanawa *hana* ‘tongue’ (*han*-a) and Poyanawa *kɨha* ‘mouth’ (*kɨ-ha*). The forms *tɨ-* ‘neck’, *han-* ‘tongue’, *kɨ-* ‘mouth’ are synchronic body-part prefixes in those languages and, thus, they can be combined with further nouns, verbs and adjectives (although verbal body-part prefixation is not productive in Iskonawa, see [7]).

Body-part roots and body-part formatives do not exhibit the same history and the same chronology. We often encounter that body-part roots are stable across languages while body-part formatives may exhibit significant cross-linguistic variation. For example, all the terms for ‘neck’ in the Pano languages in our database share the body-part root *tɨ-,* but we find substantial variability regarding the formatives recruited for the formation of the lexicalized body-part expressions (Kakataibo *tɨ-**s¸a***, Shipibo-Konibo *tɨ-**s¸u***, Matses *tɨ-**nidte***, Matis *tɨ-**tun***, Chakobo *tɨ-**puku**,* Chaninawa *tɨ-**sto*** and Kasharari *tɨ-**iwi***, among others). In some other instances, the root itself exhibits variation across the languages (cf. ‘head’, which exhibits the roots *ma-* and *βu-*). We also find full lexical innovations, for instance, *piti* ‘food’ is the word for ‘tooth’ in Chaninawa, Mastanawa, Sharanawa, Yaminawa and Nawa; while *tɨtun* ‘neck’ in Matis is ‘Adam's apple’ in Shipibo-Konibo. Finally, there are some cases of stable lexicalized forms in which both the root and the formative are shared by all or almost all the languages in our database (cf. ‘foot’, which exhibits the form *taɨ* [*ta-ɨ*?] in all the languages in our sample). At least some of these stable forms might have originated as monomorphemic words (see the discussion in 4.1).

**Figure 2.**
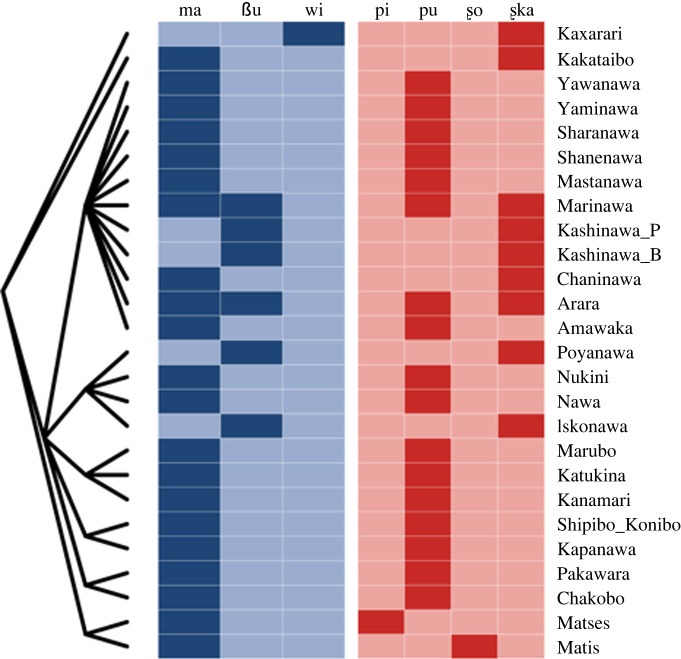
An illustration of the distribution of roots and formatives in Pano: the concept ‘head’. Roots and formatives exhibit different distributions and trigger two partially different classifications (roots appear in blue and formatives in red). The evolution of body-part expressions in Pano is diverse and suggests various morphological processes that may also have different chronologies. The internal classification of Pano languages follows Valenzuela & Guillaume [5].

As an illustration of the intricacies of body-part terms for Pano classification, table 1 features the terms for the concept ‘head’ in all the Pano varieties included in our dataset (see 2.1). There are three identifiable body-part roots associated with the concept ‘head’: *ma ‘head’, *βu ‘hair’ and *βɨ ‘eyes, forehead’. In addition, there are four formatives combined with them: *-ʂo*, *-pi*, *-pu* and -*ʂka*. Figure 2 projects the distribution of these formatives and roots in the tree presented in figure 1 (based on [5]).

**Table 1.** Forms associated with the concept ‘head’ in the Pano languages in our database.

ID	Pano language/variety	concept	form	tokens	morphemes	coding
534	Matis	head	maʂo	m a + ʂ o	head -ʂo	77 180
533	Matses	head	mapi	m a + p i	head -pi	77 184
535	Marubo	head	mapu	m a + p u	head -pu	77 76
536	Katukina	head	mapu	m a + p u	head -pu	77 76
537	Kanamari	head	mapu	m a + p u	head -pu	77 76
538	Shipibo_Konibo	head	mapu	m a + p u	head -pu	77 76
539	Kapanawa	head	mapu	m a + p u	head -pu	77 76
540	Arara	head	bapu	b a + p u	head -pu	77 76
542	Shanenawa	head	mapu	m a + p u	head -pu	77 76
543	Yawanawa	head	mapu	m a + p u	head -pu	77 76
544	Nukini	head	mapu	m a + p u	head -pu	77 76
547	Chakobo	head	mapu	m a + p u	head -pu	77 76
548	Pakawara	head	mapu	m a + p u	head -pu	77 76
551	Mastanawa	head	bapu	b a + p u	head -pu	77 76
553	Sharanawa	head	bapu	b a + p u	head -pu	77 76
554	Amawaka	head	mapu	m a + p u	head -pu	77 76
555	Nawa	head	ba:pu	b a : + p u	head -pu	77 76
556	Marinawa	head	bapu	b a + p u	head -pu	77 76
558	Yaminawa	head	bapu	b a + p u	head -pu	77 76
549	Kakataibo	head	maʂka	m a + ʂ k a	head -ʂka	77 79
552	Chaninawa	head	basakati	b a + s a k a t i	head -ʂka	77 79
541	Arara	head	*β*uʃka	*β* u + ʃ k a	hair -ʂka	81 79
545	Poyanawa	head	*β*uhka	*β* u + h k a	hair -ʂka	81 79
546	Iskonawa	head	*β*uhka	*β* u + h k a	hair -ʂka	81 79
557	Marinawa	head	ɸuʂka	ɸ u + ʂ k a	hair -ʂka	81 79
559	Kashinawa_P	head	*β*uʂka	*β* u + ʂ k a	hair -ʂka	81 79
560	Kashinawa_B	head	*β*uʂka	*β* u + ʂ k a	hair -ʂka	81 79
550	Kaxarari	head	*β*uʂkata	w ɨ + ʂ k a t a	forehead -ʂka	81 79

In this paper, we explore the history of body-part expressions in Pano aiming to quantify and understand their diachronic development. We tease apart the phylogenetic behaviour of the roots and the formatives, and we implement data analysis and clustering techniques to measure their stability. We then explore how tree-like these roots and formatives are, and investigate their potentiality for shedding light on the phylogeny of the Pano languages and for contributing to further topics in the linguistic history of Amazonia. Body-part concepts are often claimed to be basic vocabulary and therefore they are expected to be stable and conservative [8, p. 132]. The study of Pano body-part terms also constitutes a relevant contribution to the discussion of lexical stability in language. Additionally, by implementing a model where body-part roots and formatives receive independent cognancy identifiers, this study contributes to the implementation of empirical studies on partial cognacy in Amazonian historical linguistics.

## 2. Material and methods

### 2.1. Materials

**Figure 3.**
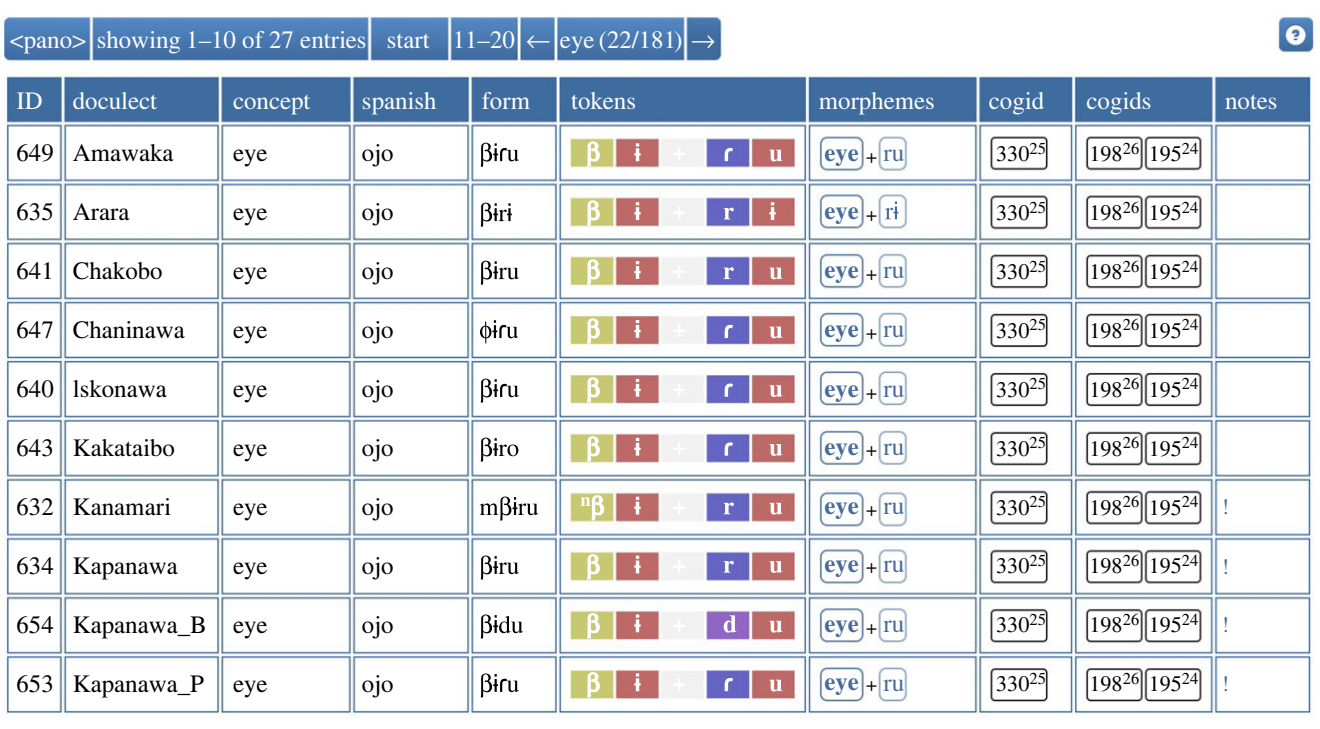
A snapshot of the Pano comparative database used in this paper.

We constructed a comparative database of a total of 26 Pano language varieties. This database contains lexical data based on concept list of 181 items (including 25 concepts related to the body). These data were automatically pre-processed by converting the tabular data that had been originally collected into long-table formats that are required by the LingPy software package [9,10] and the web-based EDICTOR tool ([11,12], https://digling.org/edictor). The conversion procedure required, among others, to standardize phonetic transcriptions by segmenting distinct sounds from each other (by adding spaces) and by using the B(road)IPA transcription system proposed by the Cross-Linguistic Transcription Systems reference catalogue ([13], https://clts.clld.org). With the help of the EDICTOR tool, the data were then annotated for partial cognancy. EDICTOR simplifies not only the annotation of partial cognates but also allows to add information on individual morphemes in the form of so-called morpheme glosses—short glosses, by which the basic meaning or function of individual morphemes can be characterized for the purpose of historical language comparison [14,15]. Figure 3 gives a snapshot of the dataset when editing it in the EDICTOR tool. In order to make the data comparable with other datasets which have been published in the past, we further converted the annotated dataset to the formats proposed by the Cross-Linguistic Data Formats initiative [16] and propose them for inclusion in the Lexibank repository [17]. Table 2 provides an overview of all languages collected in this study along with the sources we used.

**Table 2.** Forms associated with the concept ‘head’ in the Pano languages in our database.

Pano language/variety	source
Amawaka	Zariquiey's fieldwork
Brazilian Kashinawa	Camargo [18]
Chakobo	Zingg [19]
Chaninawa	Zariquiey's fieldwork
Iskonawa	Zariquiey's fieldwork
Kakataibo	Zariquiey's fieldwork
Kapanawa	Loos & Loos [20]; Loos & Loos [21]
Kasharari	Lanes [22]; Sousa [23]
Katukina	Lanes [22]; Key [24]
Marinawa	Zariquiey's fieldwork
Marubo	Fields [25]; Souza [26]
Mastanawa	Zariquiey's fieldwork
Matis	Spanghero [27]
Matses	Fleck *et al.* [28]
Nawa	Zariquiey's fieldwork
Pacahuara	East [29]
Peruvian Kashinawa	Zariquiey's fieldwork
Poyanawa	Carvalho [30]; Paula [31]
Shanenawa	Viera Candido [32]
Sharanawa	Zariquiey's fieldwork
Shipibo-Konibo	Loriot *et al.* [33]
Yaminawa	Zariquiey's fieldwork

### 2.2. Methods

The quantitative analysis was based on the organization of body-part data as feature-value vectors, in which each language of the Pano family is represented as an ordered list of binary values corresponding to the presence/absence of certain roots or formatives. To compare root and formative-based features in more detail, we divided the features into two datasets, one for roots and one for formatives. The original data were exported as a spreadsheet using a Python script to produce the mentioned representations. With this database, we perform three main quantitative calculations in order to test the influence of morphological structure of body-parts on the internal classification of the Pano family.

To serve as a first quantitative approach to the variability displayed by the morphological structure of body parts in the Pano family, using root and formative-based representations, we developed a simple exploratory analysis based on the Hamming distance [34]. The feature-value representation of each body part allows us to ask for the ‘distance’ between languages of the Pano family. We calculate distances between the language varieties in our sample as follows. For each pair of language varieties, we iterate over all of the 25 body part concepts in our data. Whenever we have data for the body part concept in both varieties, we compute the Hamming distance [34] between the binarized cognate set representations for a given concept. These individual distances are then aggregated to yield one distance for the language pair in question. These aggregated Hamming distances vary from 0 (no matching feature-value representations) to 1 (languages with the same feature-value representation). We calculated thus the aggregated Hamming distances between all language pairs, for both the root and formative-based representations. This yields distance matrices *M*(root) and *M*(formative), with 26 rows and 26 columns, in which each entry represents the aggregated Hamming distances between a pair of language varieties. With this, we compare both distributions of pairwise distances using a histogram. We used a *t*-test, implemented in *SciPy* [35], to quantify statistical differences.

Second, to assess the relative prevalence of the cognate sets in each dataset, we calculated the number of languages contained in each cognate set. Cognate sets that connect many languages will likely derive from a deep branch in the tree, and are therefore useful for recovering the deeper structure in the phylogeny. By contrast, smaller cognate sets that connect fewer languages will tend to be more recent innovations that are therefore useful for refining the fine structure of the tree topology.

Third, we applied a principal component analysis (PCA) to the distance matrix M, in order to visualize the overall similarity in the roots and formatives for the internal organization of the Pano family. This method allows us to represent languages in a two-dimensional space, in which location proximity indicates languages with a closer body-part morphological structure (in terms of roots and formatives). We used the PCA implementation of the *sklearn* library [36].

We finally calculate and plot δ scores [37,38] for body-part roots and formatives as a technique to test their tree-likeness and identify any significant difference in this regard between these two datasets. As a complement to this study basic neighbour-nets [39] were generated using SplitsTree4 program Hudson & Bryant [39] from nexus files exported from EDICTOR.

## 3. Results

### 3.1. Quantitative description of body-part morphological structure

**Figure 4.**
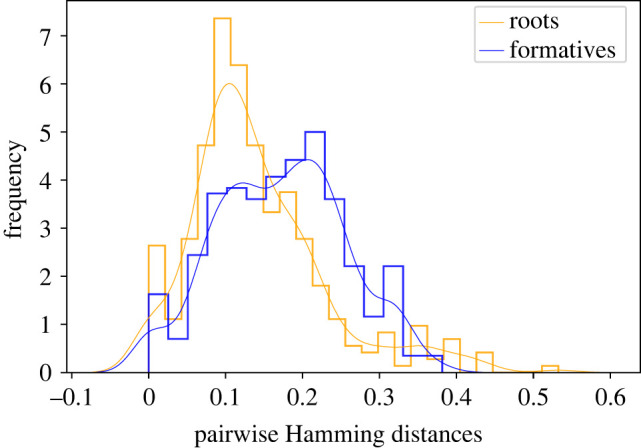
Histogram with kernel density estimate of all pairwise Hamming distances between languages as measured by the root (orange) and formative (blue) forms in the Pano family. On average the distance between languages is smaller in relation to the root dataset.

To quantitatively measure the differences between root and formative-based representations of morphological structure of the Pano family, we look at the distribution of the (average) Hamming distance between any pair of languages. On average root-based distances are shorter than formative-based distances: mean root-based distances = 0.14 (s.d. = 0.089) versus mean formative-based distances = 0.17 (s.d. = 0.0829). A Mann–Whitney *U*-test (*V* = 168888, *p* value = <0.0001) confirms this observation. Thus, based on these results, we conclude that roots are more similar lexically and phonetically across languages figure 4.^1^

**Figure 5.**
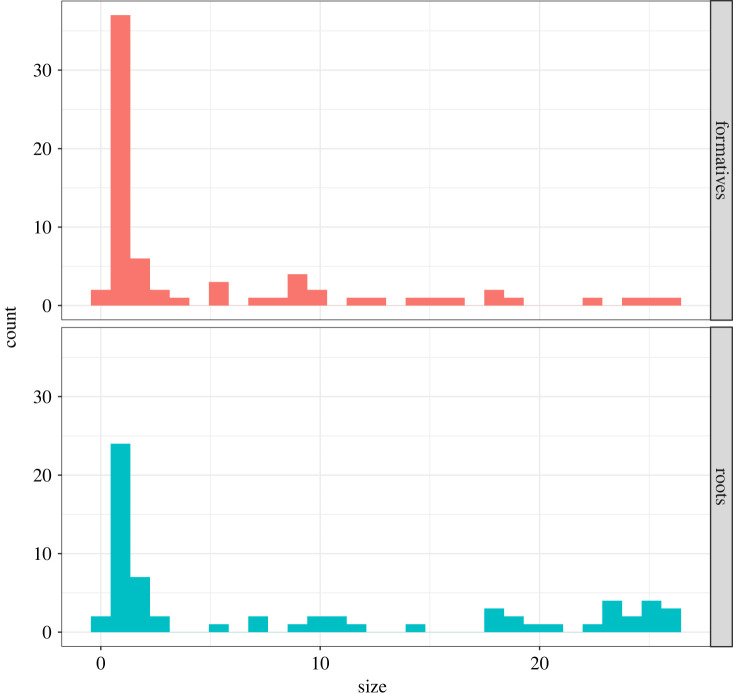
Comparison of cognate sets in roots (in turquoise, below), and formatives (in red, above). On average roots have more larger sets than formatives, and formatives exhibit a larger list of cognate sets composed of one member (over 25 instances).

Next, we quantify the size of cognate sets in the dataset (i.e. how many languages does each cognate set contain?). We find that, on average, roots connect more languages in a given cognate set: median root size = 2.5 (s.d. = 9.75) versus median formative size = 1 (s.d. = 6.84). This difference is significant under a two-tailed Mann–Whitney *U-*test (*V* = 2415, *p* < 0.0001) and is plotted in figure 5. However, this distribution is heavily right skewed, and the modal values for both roots and formatives is 1 (i.e. singletons), indicating that the mode of the cognate sets is not informative for subgrouping. Of the cognates that are informative, however, more of them are found in the roots than the formatives.

### 3.2. Low-dimensional representations of body-part morphological structure

**Figure 6.**
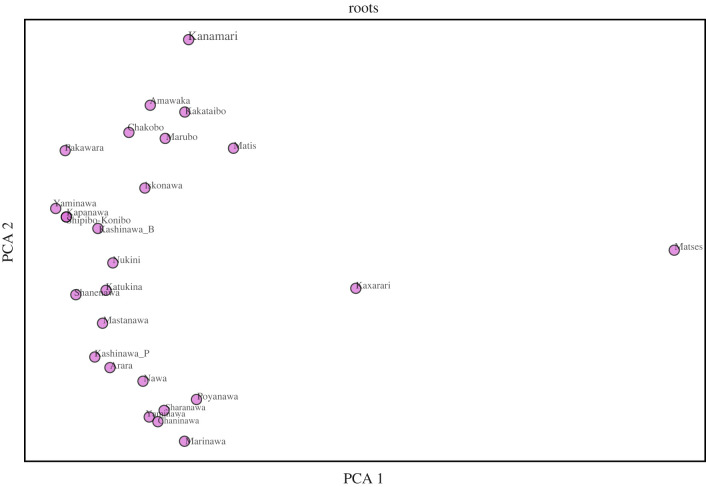
Low-dimensional representations of root (*a*) and formative-based (*b*) representations of body-part morphological structure. We applied PCA to the distance matrix M, to provide a two-dimensional representation using root and formative-based features. Root-based low-dimensional representations presents a single cluster (with a continuum-like organization regarding PCA 2 dimension) and two outliers: Kaxarari and Matses. Formative-based low-dimensional representations present one cluster, which randomly comprises languages from different branches, leaving the remaining languages in a radically discontinuous distribution.

To gain deeper insight into the internal organization of the morphological structure of body-part terminology among Pano languages, we describe the low-dimensional representation of the root and formative-based distance matrices using PCA (figure 6). The figure indicates two facts: (1) languages viewed as root-based representations are organized as a single cluster (with a continuum-like organization regarding PCA 2 values) and two outliers: Kaxarari and Matses, which, crucially, following Valenzuela & Guillaume [5], are expected to be divergent languages within the Pano family; (2) languages viewed as formative-based representations, in turn, show one cluster, which randomly comprises languages from different branches (following [5]), leaving the remaining languages in a radically discontinuous distribution.

### 3.3. Body-part roots, body-part formatives and phylogenetic signal

**Figure 7.**
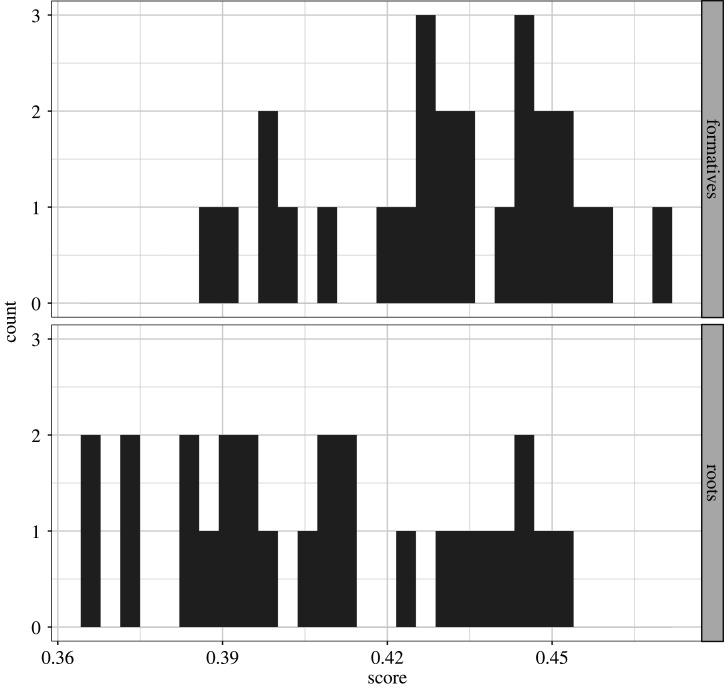
Delta scores for roots and formatives. The results show that the formatives show higher levels of non-tree-likeness.

**Figure 8.**
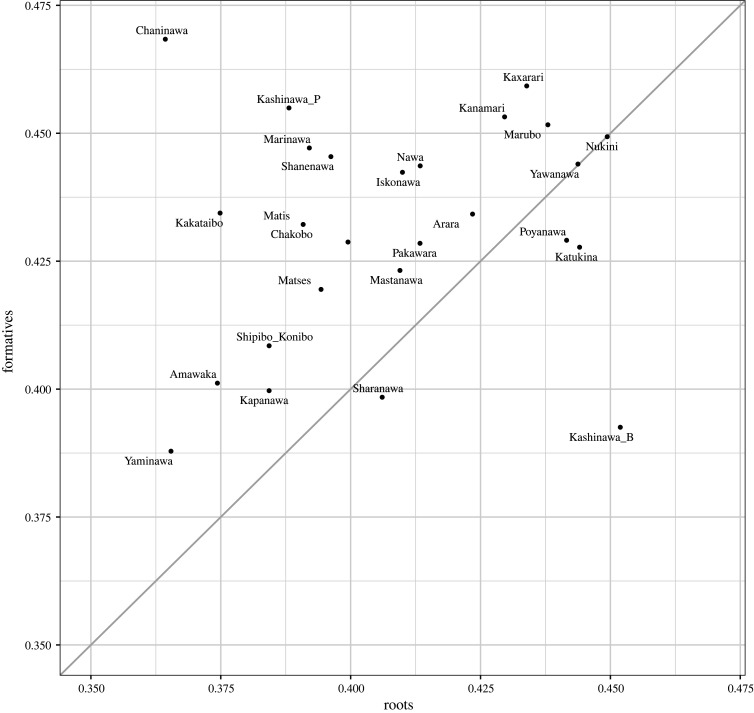
Scatterplot showing the δ scores for roots and formatives for each language. Higher δ scores are associated with more conflicting signals such as that caused by contact and diffusion. As shown by the skewed pattern of more languages above the 45° line, in most cases formatives have more conflicting signal and are therefore less tree-like than roots.

At this stage, the radically different story of roots and formatives in body-part terms becomes clear. Roots are less internally variable (i.e. more stable) than formatives (which seem to be in many cases the result of innovations in single or in small groups of languages). Furthermore, we find significant differences in how languages cluster together when they are viewed as root-based and formative-based low-dimensional representations (being the case that Pano languages as formative-based representations exhibit a saliently large internal variation or are randomly grouped together). Although these results are suggestive of some possible diachronic scenarios, it is necessary to further explore the phylogenetic behaviour of each set of forms in order to arrive at any definitive interpretation. Aiming to test the tree-likeness of body-part roots and body-part formatives we calculated δ scores [37,38] for these two datasets and plotted them. Higher δ scores indicate a less tree-like history for a given language—which could be caused by conflicting signals caused by language contact or areal diffusion of features. The histogram in figure 7 shows that Pano languages exhibit higher levels of non-tree-likeness in the formatives than in the roots. We have also included a scatter plot showing the values for each language, with formatives on vertical axis and roots on horizontal (figure 8). Languages on the 45° diagonal line have the same level of tree-likeness in both formatives and roots. Languages above the diagonal are less treelike in the formative, while below the line are languages with roots being less treelike. So Chaninawa has a high non-treelike signal in the formatives, but very low conflict in the roots, while Brazilian Kashinawa is the opposite. In general, most languages show less treelike signal in the formatives than in the roots (figure 8). We attribute this phylogenetic behaviour to the fact that formatives are the often the result of individual and parallel innovations, as discussed in §4.1.

## 4. Discussion: untangling the diachronic evolution of body-part terms

### 4.1. Toward a relative chronology of body-part terms evolution in Pano

Our quantitative experiments demonstrate that body-part roots are more conservative than body-part formatives, which are often innovative and can be attributed to specific language(s) within the family. The instability of formatives is likely behind their low tree-likeness. This, however, does not mean that the processes of body-part lexicalization postulated here happened at once. Although it is true that a good number formatives were recruited by independent languages in a relatively recent period (i.e. when Pano languages and branches were already established), some lexicalized body-part terms can be traced up to the protolanguage. The form *hana* ‘tongue’, for instance, which comes from the combination of a body-part root *han*- and the formative *-a*, is systematically attested (with predictable sound variation) in all the languages of the family. In cases like this, it is out of question that the protolanguage had already lexicalized the form *hana. At this stage, however, we cannot totally leave out the possibility that *hana ‘tongue’ was indeed originally a monomorphemic word table 3.

**Table 3.** Forms for the concept ‘tongue’ in our database.

Pano language/variety	concept	form	tokens	morphemes
Matses	tongue	ana	a n + a	mouth/tongue -a
Matis	tongue	ana	a n + a	mouth/tongue -a
Katukina	tongue	ana	a n + a	mouth/tongue -a
Kanamari	tongue	hana	h a n + a	mouth/tongue -a
Shipibo_Konibo	tongue	hana	h a n + a	mouth/tongue -a
Kapanawa	tongue	hana	h a n + a	mouth/tongue -a
Arara	tongue	ãda	ã d + a	mouth/tongue -a
Shanenawa	tongue	ana	a n + a	mouth/tongue -a
Yawanawa	tongue	ana	a n + a	mouth/tongue -a
Nukini	tongue	anã	a n + a	mouth/tongue -a
Poyanawa	tongue	anda	a n d + a	mouth/tongue -a
Iskonawa	tongue	ana	a n + a	mouth/tongue -a
Chakobo	tongue	hana	h a n + a	mouth/tongue -a
Pakawara	tongue	hana	h a n + a	mouth/tongue -a
Kakataibo	tongue	ana	a n + a	mouth/tongue -a
Kaxarari	tongue	hana	h a n + a	mouth/tongue -a
Mastanawa	tongue	ada	a d + a	mouth/tongue -a
Chaninawa	tongue	a:da	a d + a	mouth/tongue -a
Sharanawa	tongue	ada	a d + a	mouth/tongue -a
Amawaka	tongue	handa	h a n d + a	mouth/tongue -a
Nawa	tongue	a:da	a d + a	mouth/tongue -a
Marinawa	tongue	anda	a n d + a	mouth/tongue -a
Yaminawa	tongue	ada	a d + a	mouth/tongue -a
Kashinawa_P	tongue	hana	h a n + a	mouth/tongue -a
Kashinawa_B	tongue	hana	h a n + a	mouth/tongue -a
Marubo	tongue	ana	a n + a	mouth/tongue -a

The form *hana ‘tongue’ is not unique. Indeed, the form *taɨ*, which might be analysed as the combination of the body-part root *ta-* and the formative -*ɨ*, is also attested in all the Pano languages in our sample and therefore *taɨ is also unequivocally a proto-form. A similar situation is found in association with other lexicalized forms, which are attested in several languages from various branches: *βɨ-ru ‘eye’* (attested in 23 languages), *ʂɨ-ta ‘tooth’ (attested in 22 languages), *rɨ-kin ‘nose’ (attested in 19 languages), *in-a ‘tail’ (attested in 18 languages), *pɨ-i ‘feather’ (attested in 18 languages) and *ki-ʃi ‘upper leg’ (attested in 16 languages). Although some of these forms might have been originally monomorphemic (Cf. *hana ‘tongue’ and *taɨ ‘foot’), other forms like *ʂɨ-ta ‘tooth’ or *rɨ-kin ‘nose’ fully satisfy the definition of lexicalized form, and thus constitute evidence that the lexicalization process that gave rise to (some) body-part terms in Pano started relatively early.

At least some of the lexicalization processes that shape the evolution of body-part terminology in Pano happened in the protolanguage before it began to diverge. This necessarily implies that the construction in which a monosyllabic body-part root was combined with extra morphological material (i.e. what we called the formatives) was productive in a very early stage of the development of the Pano lineage. Therefore, it may have been inherited by modern Pano languages, thus providing the construction frame for future innovative lexicalizations based on conservative roots. Innovative lexicalizations seem to be abundant and this explains the non-tree-like nature of formatives, which are in constant renovation and change. This is why, as explained, while body-part roots are reflected by cognate sets that are largely invariant, body-part formatives may show a great degree of variation (cf. ‘hand’: ***më****kën* (Amawaka), ***më****dante* (Matses), ***më****bi* (Shanenawa); or ‘nose’ ***rë****kin* (Kapanawa), ***rë****xan* (Matis), ***rë****choko* (Yaminawa)).

### 4.2. Why does body-part lexicalization occur and where do the formatives come from?

Our results suggest that the construction that combines a body-part root and additional morphological material to produce a lexicalized word was already productive in the protolanguage and therefore was inherited by individual languages. Not all the lexicalization processes are equally innovative and this is why some formatives may be associated with all or a large list of languages of different branches: some formatives are retentions from the protolanguage.

A question that still remains open would be why body-part roots became combined with extra morphological material to produce new terms in the first place. This seemingly has to do with the need to refer to specific body parts. One of the challenges in the study of body-part terms has to do with the clear delimitation of their semantics ([40]: 421, [41]). Pano body-part roots seem to exhibit general meanings like ‘(related to) body-part *X’*. Their general semantics may be based on the need to implement morphological derivation to refer to more specific body-parts and related concepts. For example, in Kakataibo, the root *wɨ- ‘*(related to) eye, face’ participates in lexicalized body-parts like: ***bɨ****-ru* ‘eye’, ***bɨ****-un* ‘tear’, ***bɨ****-s¸ha* ‘rheum’, ***bɨ-****mana* ‘face, forehead, front’, ***bɨ****-bun* ‘in front of’. The lexicalization processes described here have to do with the development of new terms as a strategy to denote more specific body parts and related concepts.

A further question would then have to do with the origin of the formatives involved in these lexicalization processes. Most of these formatives are currently non-productive and exhibit an opaque semantic value. This, however, was not necessarily the case when the morphological process from which most body-part terms evolved was fully productive. Although most formatives remain semantically enigmatic, some of them can be attributed to nominal expressions, as is the case with -*kin* (< *kini* ‘hole’), -*ʂa*∼*-ʂka* (< *ʂaka* ‘skin’), *ʂu* (< *ʂuku* ‘small’), *puku* (< *puku* ‘belly’), *manan* (< *manan* ‘upper part’) and probably *-iwi* (< *iwi* ‘elongated piece of wood, tree’). Note that in some cases the formative is a reduced version of the original form, but this is not surprising, since synchronic body-part prefixes (which come from body-part roots), may reduce the form of some roots when attached to them [6]. Formatives may be fossilized forms that resulted from this morphophonemic process of root reduction. Body-part lexicalization in Pano, thus, came from body-part compounding. This explains the diversity of formatives: they come from nominal expressions in productive nominal compounding processes.

## 5. Pano body-part terminology in a broader context

### 5.1. On the origins of morphological complexity in western amazonia

It is well-known that a relatively clear-cut criterion for distinguishing Western and Eastern Amazonian languages has to do with their overall morphological profile [42]. More specifically, Amazonian languages to the West often exhibit more synthetic morphological structures with words being the result of various additive morphological processes. In turn, Eastern Amazonian languages usually exhibit analytic patterns that are closer to the ideal of morphological isolation. In this context, the question about the origin and/or development of morphological complexity in Western languages is a fundamental one. Body-part terminology shows an interesting pattern that illustrates how bound morphological elements (such as modern body-part prefixes) may arise from roots (such as old body-part roots), through processes of lexicalization, grammaticalization and reanalysis, creating a whole new paradigm of prefixes, even in suffixing languages (like Pano languages). This is in line with previous accounts of the morphological complexity of Western Amazonian languages as lexical in origin [42].

### 5.2. On parallel innovations in language classification

Since early approaches to historical linguistics, shared innovations were considered the gold standard for language clustering and tree topologies. Shared innovations are innovations that occurred in a stage that precedes language splitting, so they are likely to be inherited by the resulting linguistic varieties. Not all innovations, however, are ‘shared’ in the sense just specified. The possibility of finding the same innovation in two or more related languages as the result of independent processes is also a possibility making the task of clustering languages based on innovations a non-trivial one.

This study demonstrates that in the process of coining body-part terms in Pano, so-called body-part formatives exhibit a complex and diverse chronology and that indeed a good number of them are innovative. Nevertheless, they are less tree-like in terms of their distribution and internal structure, thus suggesting that although they may be attested in two or more languages, they do not satisfy the expectations that one would have for shared innovations. Why, then, did the process of coining body-part terms through innovative morphological combinations trigger so many instances of ‘false’ shared innovations? One possible answer to this question that may provide interesting insights into the nature of linguistic innovations may relate to the origin of the formatives. As argued in §5.2, at least some of these formatives clearly come from nouns, thus suggesting that the various instances of synchronic body-part terms were indeed nominal compounds. The crucial point here is that these compounds are not totally arbitrary. If one uses the compound *ma* ‘related to head/upper area’ + *puku* ‘belly’ which seems to be the etymology of modern term *mapu* ‘head’, the motivation may be found in the round shape that heads and bellies share. On the other hand, if one uses the compound *ma* ‘related to head/upper area’ + *xaka* ‘skin’ which seems to be the etymology of modern *maxka* ‘head’, then the motivation comes from the fact that heads are covered by skin. Such motivated compounds that lexicalized into modern terms for ‘head’ in various Pano languages can easily have happened in two or more varieties independently. As Pano body-part terminology seems to demonstrate, motivated compounds like the ones associated with some of the body-part terms in Pano seem to be more amenable to parallel development. This fully coincides with one of the major findings of this paper: according to their δ scores, Pano body-part formatives are poorly tree-like. Pano body-part terminology may, thus, be a productive domain to test hypotheses regarding the nature of grammatical innovations and their role in language classification, but also proves that δ scores may be recruited to distinguish between shared and parallel innovations in comparative databases.

### 5.3. On the phylogenetic signal of language-family-specific traits

**Figure 9.**
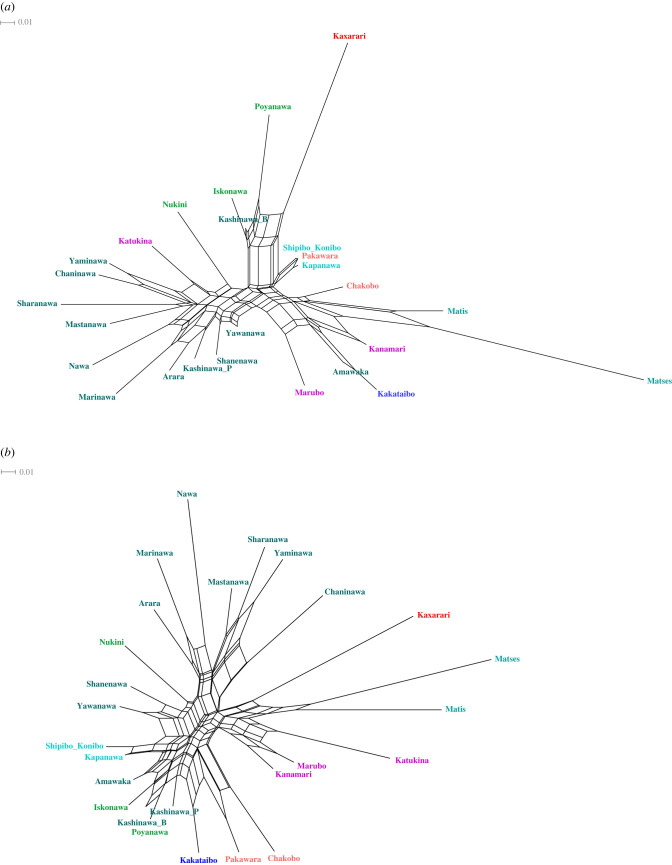
Preliminary neighbour-nets based on body-part roots (*a*) and body-part formatives (*b*). Roots succeed in reproducing the highest level of branching, by positing Matses and Kasharari as the most divergent languages, and in clustering languages in a way that quite accurately matches experts' classification, such as Valenzuela & Guillaume [5]. Body-part formatives succeed in grouping some languages from the Headwaters subgroup, but overall provide a sloppy phylogenetic structure with unclear branches. Subgroups in [5] are presented in different colours.

In this paper, we use various statistical methods to demonstrate that body-part roots are generally conservative traits that can be attributed to the protolanguage, while formatives exhibit diverse chronologies, being the case that a number of them are the result of recent and parallel innovations. Language-family-specific traits are usually ignored in quantitative phylogenetic studies. The independent analysis of body-part roots and body-part formatives led us to argue that they exhibit different levels of tree-likeness and therefore cope in different degrees to the understanding of Pano phylogeny. The use of family specific traits proves to be significant for phylogenetic studies. Our results suggest that body-part roots are expected to provide a better classification of Pano languages than body-part formatives, and crucially this is exactly the case as shown in figure 9, which features preliminary neighbour-net structures for Pano based on roots and formatives. What these neighbour-nets show is that roots succeed in reproducing the highest level of branching, in association with which Matses and Kasharari are the most divergent languages. Furthermore, roots also succeed in clustering languages in a way that quite accurately matches experts' classification such as Valenzuela & Guillaume [5]. On the contrary, although they succeed in grouping some languages from the Headwaters subgroup, body-part formatives deliver a sloppy phylogenetic structure with unclear branches (note that the subgroups in [5] are presented in different colours in the figure). The study of body-part terminology in Pano, then, contributes to language classification, by showing the relevance of introducing language-family specific traits into phylogenetic studies.

## 6. Conclusion

Here we have explored the complex diachronic story of body-part expressions in Pano languages using both quantitative methods and analytical tools from historical linguistics. Body-part expressions in Pano languages are often lexicalized forms, composed by monosyllabic bound roots and semantically opaque morphological formatives. We have demonstrated here that body-part roots and body-part formatives exhibit different diachronic trends: body-part roots are generally conservative forms that can be attributed to the protolanguage, while formatives exhibit a diverse historical signal in the sense that some are retentions from the protolanguage, but a good number of them are recent and parallel innovations in one or a few languages. The diachronic nature of the formatives is behind their highly non-tree-like nature. Based on these results, we provided a full diachronic account of body-part expressions, arguing that while body-part root are generally retentions from the protolanguage, lexicalized body-part terms, which combine roots and formatives, evolved throughout a large period of time. Lexicalized body-part expressions come from a body-part noun compounding process, which was already productive in the protolanguage (see [43]). Our results have contributed to further fields in historical linguistics and typology, by presenting a method that may efficiently tease apart shared and parallel innovations, and by showing the relevance of incorporating language-family specific traits in phylogenetic studies. Furthermore, the evolution of body-part terminology in Pano provides interesting insights into the origins of morphological complexity in Western Amazonia, by illustrating a case where its lexical origin is beyond doubt.

## Data Availability

The source code and data accompanying this study are being curated on GitHub and can be accessed from https://github.com/lexibank/panobodyparts. Data and code have also been archived with Zenodo, where they can be found at https://doi.org/10.5281/zenodo.7318438 [44].

## Endnote

1 Note that we only receive significant differences between formatives and roots when calculating the distances based on individual concepts. When representing all data by a binary vector alone, as it is common in phylogenetic approaches in linguistics, we do not find any significant differences between distances derived from roots and formatives.
